# A method for computing an inventory of metazoan mitochondrial gene order rearrangements

**DOI:** 10.1186/1471-2105-12-S9-S6

**Published:** 2011-10-05

**Authors:** Matthias Bernt, Martin Middendorf

**Affiliations:** 1 Parallel Computing and Complex Systems Group, Institute of Computer Science, University Leipzig, Leipzig, 04103, Germany

## Abstract

**Background:**

Changes in the order of mitochondrial genes are a good source of information for phylogenetic investigations. Phylogenetic hypotheses are often supported by parsimonious mitochondrial gene order rearrangement scenarios. CREx is a heuristic for computing short pairwise rearrangement scenarios for metazoan mitochondrial gene orders. Different from other methods, CREx considers four types of rearrangement operations: inversions, transpositions, inverse transpositions, and tandem duplication random loss operations.

**Results:**

An extensive analysis of the CREx reconstructions for artificial data has been presented and it is shown how the quality of the reconstructed rearrangement scenarios depends on the type of rearrangement model and additional parameter values. Moreover, a fast method is proposed to apply CREx to a large number of gene orders to find likely rearrangement scenarios and store them in a graph structure called RI-Graph. This method is applied to analyse all known metazoan mitochondrial gene orders. It is shown that the obtained RI-Graph contains many rearrangement scenarios that are described in the literature.

**Conclusions:**

The prospects and limitations of CREx have been analysed empirically and a comparison with the literature on gene order evolution highlights its benefits. The newly developed method to apply CREx to a large number of gene orders is successful in computing an RI-graph that contains many rearrangement scenarios for metazoan gene orders that have also been described in the literature. This shows that the new method is very helpful for a fast analysis of a large number of gene orders which is relevant due to the strongly increasing number of known gene orders.

## Background

Phylogenetic hypotheses are often supported by the computation of parsimonious scenarios of genome-wide rearrangement operations. Especially mitochondrial gene orders became a very fruitful source for such investigations as they are known for more than 1700 metazoan species. Furthermore they exhibit a small and usually preserved gene set [1]. Therefore, we focus in this paper on the case of sets gene orders that all have the same set of genes and do not have duplicated genes. It is assumed that several types of rearrangement operations have shaped the gene orders observed today. *Inversions* and *transpositions* are well documented [2]. Also *inverse transposition*, i.e., a transposition where the transposed part is inverted, have been found several times [1], [3-5]. Recent studies add *tandem duplication random loss* (TDRL) to the set of rearrangement operations that are relevant for mitochondrial gene order evolution [6-10]. A TDRL consists of a tandem duplication, i.e., a duplication of a continuous segment of genes such that the original segment and its copy are consecutive, followed by the loss of one copy of each of the redundant genes. The reconstruction of the evolution of gene orders for only three given gene orders is in almost all studied cases a NP-hard problem [11,12], e.g., even when only inversions are considered. This complicates studies considering combinations of different types of rearrangement operations in event based reconstruction methods, e.g., [13]. Inversions and transpositions are the most often considered genomic rearrangement operation for phylogenetic reconstruction, e.g., [14]. But ideally, all four relevant types of rearrangement operations should be regarded.

It can be found that some gene clusters are preserved during gene order evolution – be it for functional reasons or just by chance. Hence, several studies have investigated gene order rearrangement problems under the constraint that gene clusters are *preserved*. Typically, gene clusters are defined by a formal model in these studies. For gene orders without deleted or duplicated elements gene clusters are most often defined as *common intervals*[15], i.e., a set of genes that occur continuously in each of the considered gene orders. The *strong interval tree* (SIT) is a data structure to efficiently represent the set of all common intervals of two or more permutations. Based on SITs an efficient exact algorithm for computing parsimonious inversion only preserving rearrangement scenarios for two given gene orders is presented in [16]. An extension of the SIT data structure is proposed in [17] for computing shortest preserving inversion scenarios for more than two gene orders. An interesting approach to identify inversions and transpositions for pairwise gene order comparisons is to search for certain templates within the SIT [18]. In [19] the algorithm CREx (Common Interval Rearrangement Explorer) is presented which heuristically infers a preserving rearrangement scenario for two gene orders by considering all four mentioned kinds of rearrangement operations. One principle of CREx is to identify different patterns that correspond to the different types of rearrangement operations in the SIT data structure. Extensions to CREx and the TreeREx approach for automatically computing the rearrangements in a given phylogenetic tree are presented in [20]. In this paper we do an extensive study of CREx on simulated data. Even though CREx has already successfully been applied in several studies to biological data, e.g., [21] , such a systematic study is missing so far. We also propose a method for applying CREx to a large number of mitochondrial gene orders to identify likely rearrangement scenarios. The reconstructions that are obtained with the new method are stored in a so called *rearrangement inventory graph* (RI-graph). The reconstructions in the RI-graph are evaluated with a comprehensive comparison to reconstructions published in the literature.

## Materials and methods

### Gene order comparison with CREx in a nutshell

In the following we shortly introduce CREx and the SIT data structure. For more details see [19,20]. CREx compares two gene orders without duplicated or deleted genes which can be regarded as *signed permutations*, i.e., permutations with a sign (+/–) added to each element representing the orientation of the gene. A set of (unsigned) elements appearing consecutively in two gene orders is a *common interval*. Note that for the definition of a common interval the orientation and order of its elements can differ in the two permutations. Two common intervals *overlap* if they have a nonempty intersection and none is included in the other. A common interval is *strong* if it does not overlap any other common interval. The *strong interval tree* (SIT) is the graph where each node corresponds to a strong common interval and is connected to the node representing the smallest superset of genes. A node is *linear increasing* (resp. *decreasing*) if the strong common intervals corresponding to its children are in the same (resp. reverse) order in the two compared permutations and *prime* otherwise. Each node of the SIT has a *sign*. The sign of a leaf node is given by the relative orientation of the corresponding element in the permutations. The sign of a linear node is + if it is linear increasing and – if it is linear decreasing. A prime node inherits the sign of a linear parent node and is + if no such node exists.

**Figure 1:**
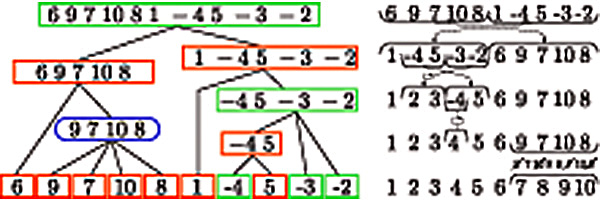
**Example SIT and CREx reconstruction.** Left: SIT of the signed permutation π = (6 9 7 10 8 1 -4 5 -3 -2) and the identity permutation; the node type is represent by its colour and shape; prime nodes are represented by a blue node with rounded corners; linear increasing (resp. decreasing) nodes are represented by red (resp. green) rectangles; right: the corresponding rearrangement scenario derived by CREx.

Consider the signed permutation *π* = (6 9 7 10 8 1 -4 5 -3 -2) and the identity permutation. In addition to the common interval {1,…, 10} and the singletons the two permutations have the following seven common intervals: {2, 3} {2, 3, 4, 5} {1, 2, 3, 4, 5}, {3, 4, 5}, {4, 5} {7, 8, 9, 10} {6, 7, 8, 9, 10}. The common intervals {2, 3} and {3, 4, 5} are not strong because they overlap. The remaining common intervals are strong and define the structure of the SIT as shown in Figure 1. The node corresponding to the strong common interval {2, 3, 4, 5} is decreasing because the child strong common intervals occur in the opposite order in *π*, i.e. in *π* they are in the order {4, 5}, {3}, {2} whereas they are in the order {2}, {3}, {4, 5} in the identity. CREx is based on the observation that the application of a single rearrangement operation leads to a pattern in the SIT which is specific for the type of the rearrangement, e.g., a transposition leads to a linear node with two linear children of opposite sign and a TDRL always leads to a prime node (unless its effect can also be described by a transposition). CREx reconstructs a short rearrangement scenario by identifying these patterns in the SIT in a specific order. Special care is taken for prime nodes with inverted elements since these cannot be explained by TDRLs only. CREx also includes the possibility for alternative scenarios, e.g., three inversions as an alternative for a transposition or several possible scenarios for prime nodes. Algorithm CREx, a tutorial, and several detailed examples are available online.

CREx represents rearrangement scenarios from *π* to *σ* in a tree data structure, defined recursively as follows. A scenario is either an ordered list of scenarios, a set of alternative scenarios, a set of pairwise commuting scenarios, or a single atomic rearrangement operation. For a rearrangement scenario from *π* to *σ* it holds that each linear lists of rearrangements generated from a traversal of the rearrangement tree where the (sub)scenarios of an ordered list are applied in the given order, the (sub)scenarios of a commuting scenario are applied in any order, only one out of alternative (sub)scenarios transforms *π* into *σ*.

For the permutation above CREx matches first the transposition pattern on the root node of the SIT and reports the transposition of the sets {1,…, 5} and {6,…, 10}. Next CREx adds the inverse transposition of the elements {2, 3} on the other side of {4, 5} to the rearrangement scenario since the corresponding pattern matches that for the node {2, 3, 4, 5}. The last pattern matching on a linear node is the inversion pattern of node {5}. Finally, the difference in the elements corresponding to the prime node add a TDRL that duplicates the elements {7, 8, 9, 10} and keeps the elements {7, 8} (resp. {9, 10}) in the first (resp. second) copy.

### Mitochondrial gene arrangement data set

The data set used in this paper is based on the 1 701 complete metazoan mitochondrial genomes in NCBI RefSeq [22], release 36 (July 2009). Since there exist several misannotations in the NCBI RefSeq data the tRNA annotation has been postprocessed with ARWEN [23] and tRNA-scan SE [24] (for more details see [25]). The gene arrangement data set consists of 185 unique gene orders having the standard set of 37 genes common to most metazoan mitochondrial genomes [1] representing 1 458 gene orders in total. The relatively small number of unique gene orders is mainly due to the fact that for most Chordata species where the gene order is known orders it is the same. But also for species in some other phyla several species have the same gene order (e.g., birds and some deep sea fishes).

### Simulated gene arrangement data sets

Each simulated rearrangement scenario is constructed by applying *r* ∈ [1 : 10] random rearrangements starting at the identity permutation of length *n* = 100. A given probability vector *p* = (*p*_I_, *p*_T_, *p*_iT_, *p*_TDRL_) specifies the probabilities that a rearrangement is an inversion (*p*_I_), a transposition (*p*_T_), an inverse transposition (*p*_iT_), or a TDRL (*p*_TDRL_). Random rearrangements, i.e., inversions, transpositions, or inverse transpositions, are chosen with equal probability from the set of all possible respective operations. A random TDRL is generated by choosing uniformly at random the duplicated interval and for each element if it is deleted in the first or second copy. We have considered six rearrangement models: (I) inversions only, (T) transpositions only, (iT) inverse transpositions only, (TDRL) TDRLs only, (IT) inversions and transpositions both with the same probability, i.e., *p* = (0.5, 0.5,0, 0), (All) all four types of rearrangement operations with *p* = (0.3, 0.3, 0.3, 0.1). Furthermore, data sets have been constructed where each rearrangement operation affects the order of at most *w* ∈ {10, 20,…, 100} genes. For each combination of rearrangement model, *r*, and *w*, 1000 data sets have been simulated, i.e., altogether 600 000.

Each pair of the identity permutation with one of the generated permutations has been used as input for CREx. Let *S* be the set of rearrangement operations in the simulated scenario and *C* the rearrangement scenario computed by CREx. The quality of the CREx scenario is measured by precision = (|*S* ∩ *C*|)/|*C*| and recall = (|*S* ∩ *C*|)/|*S*| for |*S*| ≠ 0 ∧ |*C*| ≠ 0 (if |*S*| = |*C*| = 0 precision and recall are 1, if |*S*| = 0 ∧ |*C*| ≠ 0 or |*S*| ≠ 0 ∧ |*C*| = 0 precision and recall are 0). The “intersection” operation between *S* and *C* is computed with a corresponding function provided by TreeREx [20] which determines the longest common suffix between *S* and *C* or more precisely between any pair of the linear lists of rearrangements generated from a traversal of the tree representations of *S* and *C*.

### Building the rearrangement inventory graph

Let Π = {*π*_1_,…, *π_k_*} be a set of gene orders. A directed graph *G* = (Π, *E_l_* ∪ *E_p_*) *—* called the *rearrangement inventory graph* (RI-graph)— is defined on the set of nodes Π where the edges represent rearrangement scenarios reconstructed by CREx. Recall, the aim is to represent only those rearrangement scenarios that can be considered as likely candidates for rearrangements that might have happened during evolution. Therefore, edge set *E_l_* contains only edges between two permutations *π_i_* , *π_j_* for which the corresponding SIT has only linear nodes. Furthermore, for at least one of the two permutations, e.g., *π_i_* , it must hold that there does not exist a third permutation in Π which has a smaller distance to *π_i_* than the distance between *π_i_* and *π_j_*. The distance *d* between two permutations is defined as the length of the corresponding CREx scenarios (in case of alternative scenarios the shorter alternative is regarded). More exactly, two nodes *π_i_*, *π_j_* ∈ *V*, with 1 ≤ *i* ≠ *j* ≤ *k* are connected by an edge in *E_l_* if, i) the SIT for {*π_i_*, *π_j_*} is linear, and ii) either there is no gene order *π_h_* ∈ Π, with *i* ≠ *h* ≠ *j* such that the SIT for {*π_h_*, *π_j_*} is linear and *d* (*π_i_*, *π_h_*) <*d*(*π_i_*, *π_j_*) or the analogous statement holds for *j* instead of *i*. Observe, that the scenarios corresponding to edges in *E_l_* do not contain any TDRL operation. Hence, edge set *E_p_* is introduced to consider likely rearrangement scenarios containing a TDRL operation. Since TDRL operations are considered as rare, scenarios that have more than one TDRL operation are not considered for introducing an edge to *E_p_*. Formally, there exists an edge (*π_i_*, *π_j_*) ∈ *E_p_* if, i) the rearrangement scenario from *π_i_* to *π_j_* includes exactly one TDRL, ii) *π_i_* and *π_j_* are in different connected components of the graph (Π, *E_l_*), and iii) . The second condition states that a scenario with TDRL operations is not considered when the corresponding permutations are connected in (Π, *E_l_*), i.e., they are already related to each other with a likely scenarios not requiring TDRL operations.

### Representation of the rearrangement inventory graph

The nodes of the RI-Graph are shown as rectangles labelled with the accession number of one representative species and the number of species with the same gene order in parentheses if larger than one. Edges are coloured with respect to the represented rearrangement scenario such that the fractions of TDRLs, inversions, and transpositions correspond, respectively, to the intensity of the colours red, green, and blue. For the purpose of colouring an inverse transposition is counted as an inversion plus a transposition. The direction of a scenario that contains a TDRL is indicated by a directed edge. Each edge is labelled with the corresponding unique identifiers of the rearrangements that are predicted by CREx. An index of the identifiers of all predicted rearrangements is given in Additional file 1. The layout of the graph was done manually starting from an initial layout computed with Graphviz (http://www.graphviz.org).

## Results and discussion

### Empirical analysis of CREx reconstructions

An empirical analysis of the quality of the reconstructions of CREx on the simulated data is presented in the following for the different models of genome rearrangement. Clearly, it can not be expected that CREx is able to reconstruct the simulated scenarios for a large number *r* of rearrangement operations. The reason is that there exist too many possible rearrangements and also the shortest possible rearrangement scenario between two simulated permutations is not necessarily the simulated one. The hope is, that for a small number of rearrangement operations CREx can deliver good reconstructions. In that case such reconstructions might be useful as a basis for the analysis of the phylogeny even for a large number species (as done in the second part of the results section) because CREx is also very fast. For the 600 000 reconstructions CREx needed 21 min 54 s on a laptop with a 2.0 GHz processor, i.e., one reconstruction in ≈ 10^–3^ s on average.

#### Reconstruction quality

**Figure 2:**
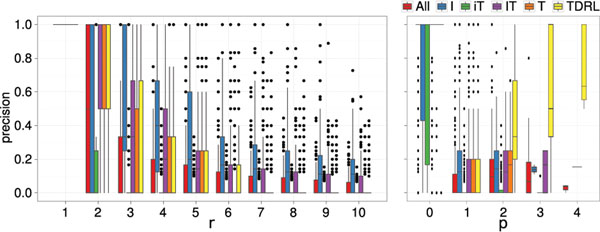
**Reconstruction Quality of CREx.** Precision of CREx reconstructions for the simulated data sets for the different rearrangement models; left: precision for different rearrangement numbers r ∈ [1 : 10]; right: precision for different numbers of prime nodes p of the SIT.

Boxplots of the precision of the CREx reconstructions for different numbers of rearrangement operations *r* are shown in Figure 2 (left). The corresponding plots for the recall values are omitted (see Additional File 1), because they are very similar to the corresponding precision values, i.e., the average precision and recall values differ by ≤ 0.05.

It can be seen that for *r* = 1 the correct scenario was always found by CREx. For *r* = 2 and I (resp.. TDRL) the majority of the rearrangement scenarios. i.e., 686 (resp. 710), has been reconstructed correctly. For the rearrangement models IT and All the correct scenario has been reconstructed for considerably more than one third of the data sets (425 and 435). A correct scenario has been reconstructed for less than one third (306 resp. 211) of the data sets for T and iT. For a large number of rearrangements (*r* > 9 and I, *r* > 8 and IT, *r* > 5 and TDRL, All, *r* > 4 and T, *r* > 3 and iT) a reconstruction with a recall of one could not be done for a single data set. But often at least a part of the rearrangement scenario could still be reconstructed, e.g., for at least 678 of the data sets generated with I for *r* ≤ 10 and the majority of the IT (T, iT, TDRL) data sets for *r* < 6 (*r* < 4, *r* < 2, *r* < 3).

Thus, for small values of *r* the quality of the reconstructed scenarios is good, but decreases for larger values of *r*. CREx is able to reconstruct at least a part of the simulated rearrangement scenario for many data sets with medium values of *r*. This is an interesting observation, because a partially correct reconstruction might suffice for the correct operation of TreeREx [20] which is based on determining common suffixes in rearrangement scenarios leading to a permutation which has been determined with CREx. The results are apparently better when no transpositions or inverse transpositions are applied. Although these first results for simulated data seem not to be very promising, additional biologically relevant criteria and constraints to the rearrangement models can be identified where CREx’ performance is better.

#### Sensitivity to strong interval tree structure

In this subsection it is shown that the reconstruction quality depends to a large extent on the properties of the SIT corresponding to the pair of permutations analysed by CREx. Figure 2 (right) shows the precision for the simulated data sets in relation to the number of prime nodes in the corresponding SITs. Plots for recall are given in Additional File 1 (difference of the average values ≤ 0.06).

The results for the TDRL model differ from the results of the other rearrangement models in an increasing reconstruction quality for larger values of *p*. This is discussed first. The cases with *p* = 0 generated with TDRLs correspond to one of the rare cases (155*x* for *r* = 1 and 19*x* times for *k* = 2) where the random TDRL has the same effect as a transposition. If the random TDRLs do not overlap they create separate prime nodes, i.e., *k* = *p* (the inverse does not necessarily hold). Such cases can easily be reconstructed by CREx. This is confirmed by the observation that for *p* = *k* the precision and recall values have been 1 in all but two cases for *k* = 2. Furthermore, the fraction of data sets with *k* = *p* increases with *p*, from 0.11 for *p* = 1 to 0.6 for *p* = 4. This explains the difference of the results for large *p* for the TDRL model. The reconstructed scenarios of CREx are mostly correct when the SIT has no prime node. This holds for all rearrangement models. But, when the SIT has a prime node, a large fraction of the reconstructed rearrangements is not correct.

There are 10833 random data with prime node free SIT. For 8101 (≈ 75%) of these data sets the CREx reconstruction is correct and at least partially correct for 9 616 (≈ 89%). For the remaining 49 167 data sets, which have a prime node in the SIT, the CREx reconstruction is correct only 2128 (≈ 4%) times and at least a part of the reconstructed scenario was correct for only 17 741 (≈ 36%) of these data sets. For *r* = 1 the SIT is, except for TDRL, always prime node free. But also for the data sets with *r* > 1 the absence of prime nodes in the SIT is still a good indicator for the quality of CREx’ reconstructions. This is, ≈ 53% correct (resp. ≈ 79% partially correct) reconstructions for data sets with prime node free SIT compared to only ≈ 7% (resp.. ≈ 35%) when the SIT has prime nodes.

The presented results clearly show that the absence of prime nodes in the SIT is a good indicator for the quality of the rearrangement scenario reconstructed by CREx. This is remarkable because pairwise comparisons of metazoan mitochondrial gene arrangements often correspond to prime node free SITs and most of the gene orders take part in at least one such comparison (see Additional File 1).

#### Different rearrangement sizes

**Figure 3:**
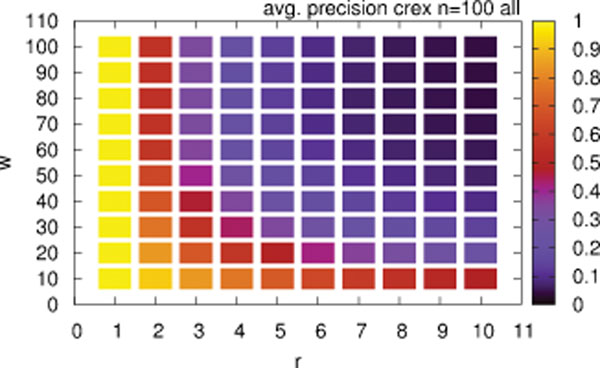
**Reconstruction Quality of CREx for Different Rearrangement Sizes.** Average precision of CREx for simulated data sets for different numbers of rearrangements *r *and different numbers of affected elements *w*; averages are computed over the results of all five rearrangement models for each combination of *r* and *w*.

The probability of the rearrangements may depend on additional properties in real world scenarios, e.g., short rearrangement operations are found more often [26] (and references therein). In the following the influence of the length (measured as the number *w* of influenced genes) of the rearrangement operations on the reconstruction quality of CREx is analysed (Figure 3). It can be seen that the reconstructions of CREx are of much better quality for smaller values of *w*. When comparing the unrestricted case (*w* = 100) and the case *w* = 10 an improvement of the average precision and recall of at least 0.44 was measured for all values *r* > 2. Of course the structure of the SIT depends on the applied rearrangements. For short rearrangement operations the number of simulated data sets without prime nodes increases, e.g., 53 819 data sets for *w* > 50 and 88558 for *w* ≤ 50 are prime node free. Thus, the effects of increased reconstruction quality in the case of prime node absence and shorter rearrangement operations are not independent. See [27] for a formal study of the relation of rearrangement length and the properties of the generated gene clusters. The presented results indicate that the quality of CREx’ reconstructions is improved for gene orders that evolved with short rearrangements. Clearly, since there is no accepted model for mitochondrial gene order evolution this does not necessarily imply an improved reconstruction quality of CREx for mitochondrial gene orders.

### Inventory of metazoan mitochondrial gene order rearrangements

The RI-Graph has been computed for the set of all 185 unique complete Metazoan gene orders. The computation needed 30s on a laptop with a 2.0 GHz CPU. The 185 nodes of the resulting RI-Graph are organised in several connected components. Most of the connected components are small: 29 nodes are singletons, nine components contain two nodes, six components contain three nodes, there exists one component of size five, and one of size eight. Additionally, there are two large components containing together more than half of the nodes. One of these components has 45 nodes and represents, with the exception of one Priapulid, gene orders from arthropod species. The other large component contains 62 nodes which correspond to gene orders of Chordata plus one Xenoturbellida and one Hemichordata.

In the following three large connected components of the RI-Graph are analysed in more detail. A more comprehensive analysis of the results is given in [25]. Note, that the study presented in the following is not intended to be phylogenetically conclusive.

#### Mollusca

The mollusc gene orders are organised in five connected components of size greater than one and three singletons nodes (*S. lobatum*, *G. eborea*, and *P. dolabrata*).

**Figure 4:**
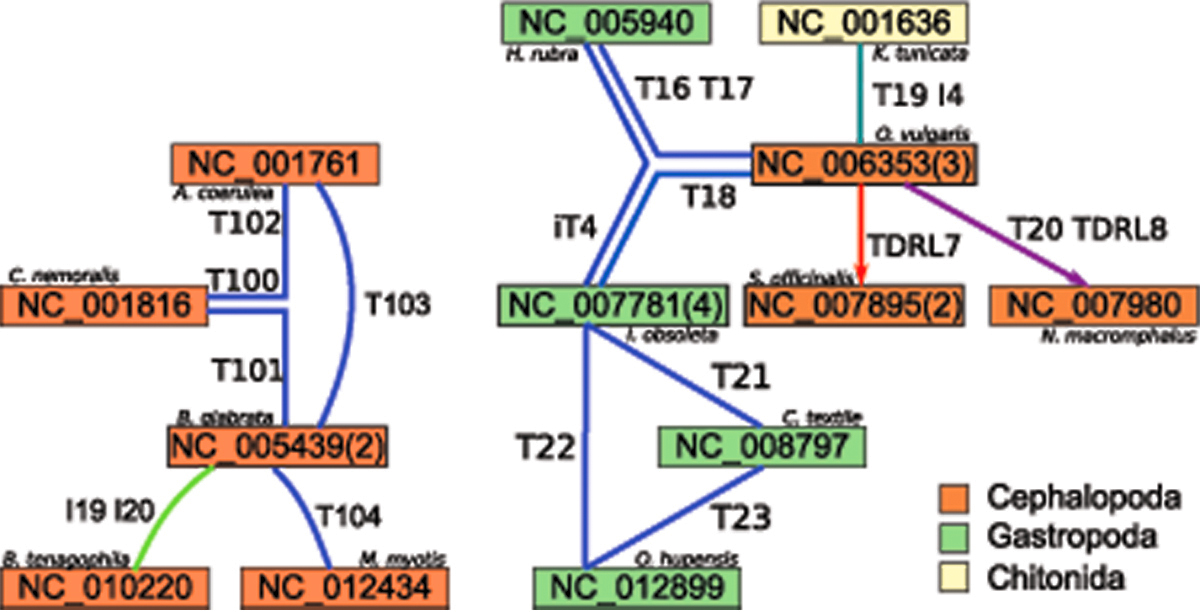
**Large Mollusca Components.** The two larger connected components including mitochondrial gene orders from Mollusca; left) five Gastropoda; right) four Gastropoda, one Chitonid, and three Cephalopoda

Scenarios for five gastropod gene orders are given in the connected component shown in Figure 4 (left). I19 and I20 are presumably caused by tRNA annotation errors since ARWEN and tRNAscan already report five differently oriented tRNAs for NC_01022. Hence, we exclude *B. Tenagophila* from the following discussions. The transpositions T100 and T102-104 are given in [28]. Transposition T101 suggest a previously unreported alternative scenario. Assuming the tree topology given in [28] a parsimony analysis of the presented results suggest that the gene arrangement of *C. nemoralis* can not be ancestral. In addition to the three other gene orders the gene arrangement separated by T100 from *C. nemoralis*, by T101 from *B. glabrata*, and by T102 from *A. coerulea* is a putative ancestral gene order. But note that every unrooted tree topology including the four species is equally parsimonious.

The largest connected component of mollusc gene orders (right part of Figure 4) contains gene orders from Gastropoda, Cephalopoda, and one Chitonid. Transpositions T16-19 and inversion I4 are reconstructed as reported in [29], inverse transposition iT4 is given there as two separate inversions. Note that the CREx scenarios between the gene orders of *O. vulgaris*, *H. rubra*, and *I. obsoleta* have many common rearrangements. This proposes the gene order separated by iT4 from *I. obsoleta*, by T18 from *O. vulgaris*, and by T16, T17 from *H. rubra* as the ancestral gene arrangement for at least two of the three gene arrangements (most likely of the two Gastropoda). Assuming any of the three gene orders as ancestral would not be parsimonious. TDRL7 is presented in [6]. The scenario to *N. macromphalus* is proposed as “transposition of two large blocks and transposition of F” instead of TDRL8, but transposition T20 is reported equivalently in [30].

#### Arthropoda

**Figure 5:**
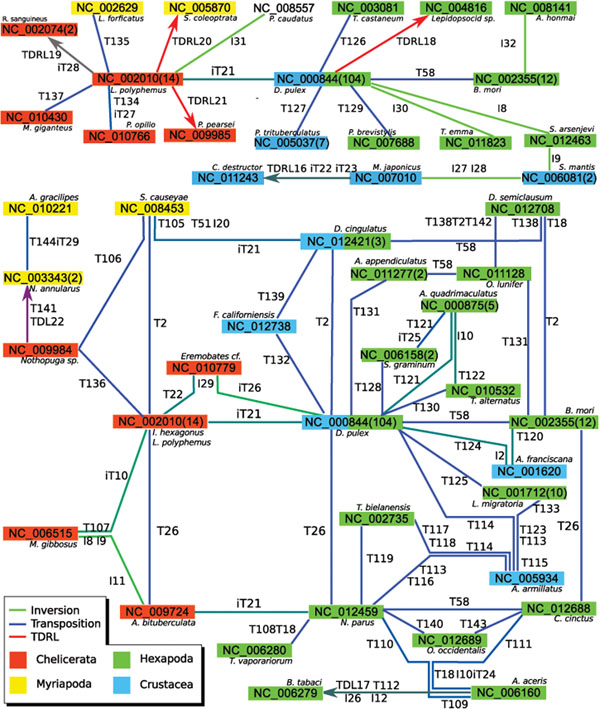
**Large Arthropoda Component.** Large connected component of arthropod mitochondrial gene orders; top: nodes which are only adjacent to nodes NC 002010, NC 000844, and NC 002355 plus chain of 4 nodes adjacent to NC 000844; bottom: rest of the component.

The gene orders of the Arthropoda are clustered in nine connected components. The component containing 45 of the 77 unique arthropod gene orders is shown in Figure 5. The subgraph defined by the nodes NC_002010, NC_000844, and NC_002355 and nodes which are only adjacent to one of these three nodes (plus one chain of nodes — NC_011243, NC_007010, NC_006081 — connected to NC_000844) is given in the upper part of the figure. The remaining part of the connected component is presented in the lower part. The component contains two unique gene orders representing Hexapoda and Crustacea (Pancrustacea). That is, NC_000844 represents 90 Hexapod and 14 Crustacean gene orders and node NC_012421 represents gene orders of two Hexapoda and one Crustacea. The gene order corresponding to node NC_000844 is considered to be the ancestral Pancrustacean gene order [31] and the gene order of *L. polyphemus* (NC_002010) is regarded as the ancestral arthropod gene order [32].

Several rearrangements that have been found occur several times between different pairs of permutations. Therefore, some of these rearrangements can be found several times in the literature. These are for example i) T26 is a swap of *I* and *Q*, e.g. [33,34], ii) iT21 is an inverse transposition of *L2*[1,35], iii) T58 is a transposition of *M* and the pair *I*, *Q *[36], and iv) T2 is swap of *P* and *T*, e.g., [8,33]. These rearrangements are of great interest because they have been found several times between or within different taxonomic groups and may be examples of convergent or synapomorphic rearrangements, as discussed in [37]. The results presented here will be of great help to investigate such problems.

First, some rearrangements shown in the lower part of Figure 5 are analysed. Using the presented method CREx automatically found TDRLs 16,18,20, and 21 which are striking examples of the tandem duplication random loss model of genome rearrangement. TDRLs 20 and 21 have been presented in [8,9] differing only by the exclusion of a single tRNA from the TDRLs favouring an additional transposition of the tRNA in both cases. This is because the control region is used in these studies as additional evidence. It is interesting that seven (five) transpositions would be necessary for an alternative explanation of TDRL20 (resp. TDRL21). Three TDRLs are needed for the rearrangements in the opposite direction in both cases. Note that [38] postulated “at least 10 translocations” for the rearrangements leading to *C. Coleoptrata*. In [39] tandem duplication random loss was discussed as a possible cause of the rearrangement in the undescribed Lepidopsocrid species, but the actual TDRL (18) is given here for the first time. In [40] the gene orders of *C. destructor* and *D. pulex* have been compared and different positions for eleven genes have been noticed where for two genes inversion is involved. “For nine of the translocations, the ’duplication/random loss’ mechanism” was suggested to be plausible and “a minimum of five independent duplication/random loss events” have been postulated. The automatic reconstruction using CREx – iT22, iT23, TDRL16 – matches the description perfectly. But potential misannotation of the *V* and the rRNAs in *S. arsenjevi*, *S. mantis*, and *M. japonicus* make further investigations necessary. Instead of TDRL19 two transpositions are proposed in [3], but iT28 is given equivalently. In [10] the gene orders of *L. polyphemus* and *N. annularus* have been compared and a tandem duplication non-random loss rearrangement and a transposition have been proposed. That is in each copy only genes on the same strand are lost (with one exception). CREx reconstructs the same rearrangements (T141 and TDRL22) but with an intermediate step via the gene order of *Nothopuga* sp. by transposition T136 [9]. T144 and iT29 are reconstructed as in [5], where it was speculated that the transposition is derived from the pre-”non random loss” tandem duplicated gene arrangement that gave rise to the *N. annularus* gene order.

#### Rearrangement counts

Table 1 shows the numbers of rearrangements operations found in the analysed connected components of the RI-Graph, i.e., without the components containing Deuterostomia. Note, that the total number of unique rearrangements can be less than the sum of the corresponding numbers of known, different and new rearrangement operations, because the same rearrangement can occur several times in the RI-Graph and might be counted differently for different pairs gene orders. The strength of the proposed RI-Graph approach is shown by the fact that most of the proposed rearrangements are likely to be correct in the sense that they are in agreement with the literature. Within the analysed components a high number of transpositions have been found. This is in agreement with the results presented in [4]. Also [37] manually identified 43 transpositions within the presented 67 comparisons of mitochondrial gene orders of Hymenoptera. For two bacterial genomes also a high number of transpositions was reported in [41].

**Table 1:** Rearrangement Type Counts

	#	known	diff	new
I	44 (26)	13 (9)	14 (8)	17 (15)
T	109 (71)	70 (42)	14 (11)	25 (24)
iT	18 (14)	10 (8)	4 (2)	4 (4)
TDRL	16 (16)	7 (7)	3 (3)	6 (6)

Number of rearrangement operations found in the analysed connected components of the RI-Graph; #: total number of rearrangements in the graph; known: rearrangements in agreement with the literature; diff: rearrangements reported differently in the literature or which are caused by annotation errors; new: rearrangements that have not been found in the literature; in parentheses: number of unique rearrangements

We would like to stress the point that these findings are apparently in disagreement with weighting schemes which put more weight on transpositions, e.g., [13,42]. This is done to avoid a bias favouring transpositions since they are argued to be “observed much less frequently than inversions in many evolutionary contexts” [42] and also [43]. The results presented here indicate that it is necessary to re-examine — at least for intra phylum comparisons — the weighting schemes that are typically used in the literature for the analysis of mitochondrial gene order rearrangements.

## Conclusions

The quality of the mitochondrial gene order rearrangement scenarios reconstructed with CREx has been analysed in an extensive study on artificial gene orders that have been simulated with different rearrangement models. The four type of rearrangement operations - inversion, transposition, inverse transposition, and tandem duplication random loss (TDRL) — that are relevant for mitochondrial gene order evolution have been considered. It was shown that the absence of prime nodes in the strong interval tree (SIT) and a not too large number of genes involved in the rearrangement operations are good criteria indicating reliable CREx reconstructions.

Based on the simulation results a method has been proposed that can be used for automatically analysing a large number of mitochondrial gene orders in order to find a likely subset of scenarios from pairwise gene order comparisons. The found scenarios are stored in an RI-Graph data structure. The proposed method was applied to all known metazoan mitochondrial gene orders resulting in an RI-Graph where most gene orders within the same phylum are connected. The potential of the new method was shown by the large agreement with the literature on gene order rearrangements within the Protostomia.

Mitochondrial gene arrangement data are mostly still analysed manually by biologists [37]. Such manual analyses are valuable and indispensable, but the handling of the huge amount of available data is at least tedious and may also be considered as impossible. Therefore, usually only a very small number of gene arrangements is compared (with notable exceptions [9,37]), only a subset of the genes is evaluated (usually tRNAs are excluded), or only a part of the arrangements is compared (usually all species are compared to a putative ancestral gene order [37], or based on a phylogenetic tree [9]). In this way the phylogenetic signal which is or is not contained in gene order data can not be properly analysed or important alternative rearrangement scenarios may be missed. The new method facilitates the automatic analysis of gene orders and the reconstruction of rearrangement scenarios. Within less than a minute the results for the complete mitochondrial data set can be computed. It was shown here that CREx allows for a comprehensive analysis of the rearrangements, within the connected components, solving some of the problems mentioned above, in an unprecedented and efficient way. Furthermore, the possibility to identify possible ancestral states and cases of convergent evolution has been indicated. For future work it is promising to refine and extend the proposed method, e.g. the inclusion of methods for the handling of missing genes or duplicates.

## Competing interests

The authors declare that they have no competing interests.

## Authors contributions

MB designed and implemented the algorithms, performed the analyses of the artificial and mitochondrial data sets. MM provided design suggestions for the algorithms as well as the analyses. MB and MM conceived the project and wrote the manuscript.

## Supplementary Material

Additional File 1 **PDF with Supplementary Material** For the simulated data the plots for recall and the number of prime node free data sets for the used rearrangement models are given. The numbers of prime node free comparison for the mitochondrial data set are listed. The remaining connected components and the rearrangement scenarios for all connected components – excepting the Chordata which have not been analysed in detail – are given. Click here for file
