# Supplementary material for: A method for computing an inventory of metazoan mitochondrial gene order rearrangements
Source: BMC Bioinformatics. 2011 Oct 5;12(Suppl 9):S6. doi: 10.1186/1471-2105-12-S9-S6 (PMC3283314; doi:10.1186/1471-2105-12-S9-S6)
Supplement: Additional File 1 — PDF with Supplementary Material For the simulated data the plots for recall and the number of prime node free data sets for the used rearrangement models are given. The numbers of prime node free comparison for the mitochondrial data set are listed. The remaining connected components and the rearrangement scenarios for all connected components – excepting the Chordata which have not been analysed in detail – are given. [file 1471-2105-12-S9-S6-S1.pdf]

Supplement to: A Method for Computing an Inventory of Metazoan Mitochondrial Gene Order Rearrangements

Matthias Bernt*[1], Martin Middendorf[1]

[1]Parallel Computing and Complex Systems Group, Institute of Computer Science, University Leipzig, Germany

Email: Matthias Bernt*- bernt@informatik.uni-leipzig.de; Martin Middendorf - middendorf@informatik.uni-leipzig.de;

*Corresponding author

Simulation Results

Reconstruction Quality

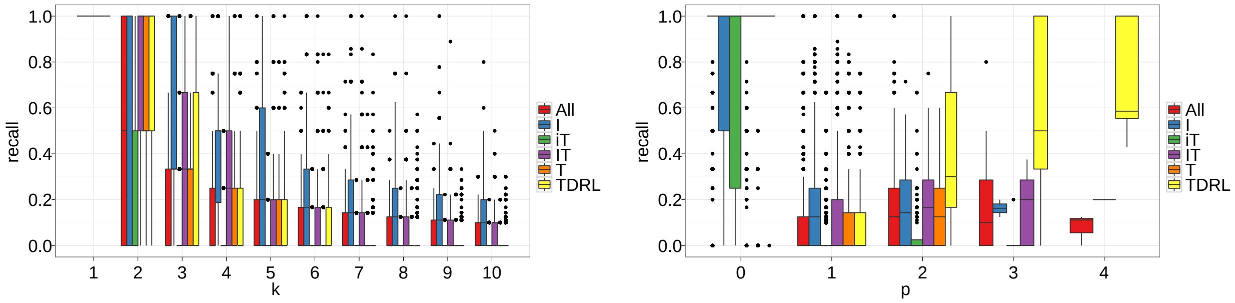

Figure 1 – Recall of CREx reconstructions for the simulated data sets for the different rearrangement models; left: recall for different rearrangement numbers $r \in [1 : 10]$; right: recall for different numbers of prime nodes p (right) of the strong interval tree

Sensitivity to Strong Interval Tree Structure

The amount of simulated data sets, which have at least one prime node in the corresponding strong interval tree, is given in Table 1.

Different rearrangement sizes

r	I	T	iT	TDRL	IT	All
1	0	0	0	845	0	80
2	0	574	425	981	399	477
3	248	912	843	1 000	714	812
4	538	987	978	1 000	902	947
5	766	1 000	994	1 000	971	986
6	876	1 000	999	1 000	995	998
7	954	1 000	1 000	1 000	997	1 000
8	979	1 000	1 000	1 000	999	1 000
9	992	1 000	1 000	1 000	1 000	1 000
10	999	1 000	1 000	1 000	1 000	1 000

Table 1 – Number of simulated data sets which have at least one prime node for different $r \in [1 : 10]$ and rearrangement models; 1 000 data sets per combination; I, T, iT, TDRL, IT, and All as defined in subsection "Simulated Gene Arrangement Data Set"

	act	nac	cho	ehx	cru	hex	cmy	art	abm	np	all
l %	68.8	59.5	60.2	3.6	16.3	51.3	9.4	19.6	9.5	7.8	10.2
L %	89.3	94.9	91.8	36.4	75.0	83.3	59.3	71.4	65.2	66.7	71.9

Table 2 – Properties of the SITs for all pairs in the mitochondrial data sets; l: percentage of gene order pairs having no prime node; L: percentage of the gene orders being in at least one pairwise comparison without a prime node; act: Actinopterygii; nac: non Actinopterygii; cho: Chordata; ehx: Echinodermata, Hemichordata, and Xenoturbellida; cru: Crustacea; hex: Hexapoda; cmy: Chelicerata and Myriapoda; art: Arthropoda; abm: Annelida, Brachiopoda, Echiura, Mollusca, and Sipuncula; np: Nematodes and Platyhelminthes

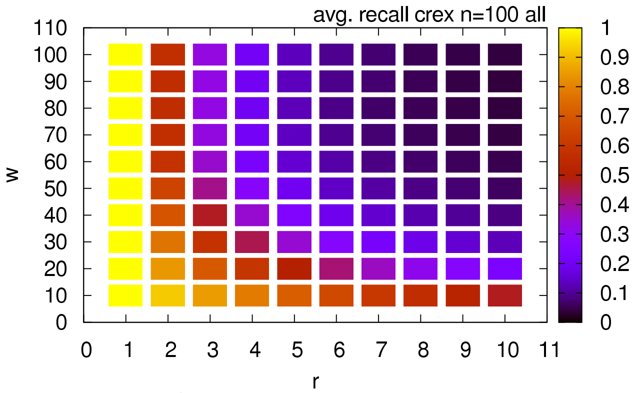

Figure 2 – Average recall of `CREx` for simulated data sets for different numbers of rearrangements r and different numbers of affected elements w; averages are computed over the results of all five rearrangement models for each combination of r and w

1 RI-Graph and Lists of Rearrangement Scenarios

In the following the remaining connected components and the rearrangement scenarios for all connected components – excepting the Chordata which have not been analysed in detail – are given. Each of the following sections presents the components and the corresponding rearrangements for different phyla. The rearrangement scenarios

- from Figure 4a) and 4b) of the main text are given in Figures 12 and 13
- from Figure 5 of the main text are shown in Figure 24,
- from Figure 6 of the main text are given in Figures 25 to 28
- from 5 and 6 of the main text which include iT21, T2, T58, and T26 are given separately in Figures 29 to 31.

Different rearrangement scenarios are separated by a horizontal line. In order to save space, each rearrangement scenario is presented as follows. Let π and σ be two gene orders and $\rho_1, \ldots, \rho_k$ be a rearrangement scenario from π to σ. The rearrangement scenario is given in k lines where line i, with $1 \leq i \leq k$, presents the strong interval tree of the two permutations rearranged by the first $i-1$ rearrangements of the scenario. Hence, reading the leaves of the tree from left to right gives the permutation after the first $i-1$ rearrangements are applied. In the strong interval tree of the i-th line the rearrangement ρ_i is marked (the colours are as is Section 1.5). The result of the last rearrangement σ is not shown. The unique identifier of the rearrangement is given on the left. If possible a citation is given on the right where the corresponding rearrangement has already been documented. For a detailed discussion see [1]

Rearrangements which are compliant to the literature are marked with •. Differently reconstructed rearrangements or rearrangements that may be caused by annotation errors are marked with •. Rearrangements that have not been found in the literature are marked with ◦.

1.1 Echinodermata

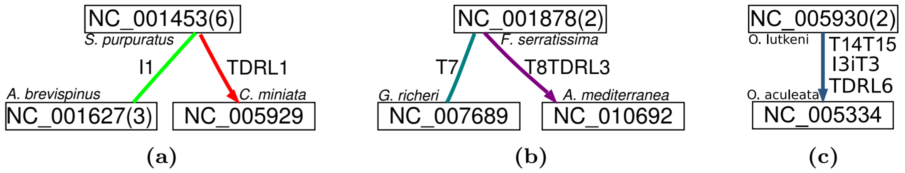

Figure 3 – Connected components of size greater than one including gene orders from Echinodermata a) *Echinoidea* (top node), *Asteroidea* (bottom left), and *Holothuroidea* (bottom right node and one in the top node) b) *Crinoidea*, c) *Ophiuroidea*

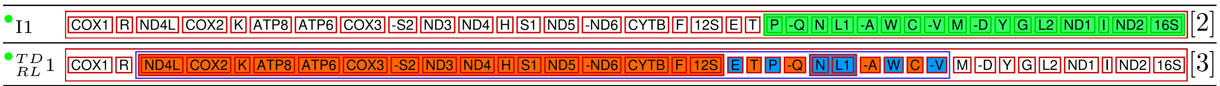

Figure 4 – Rearrangements from the connected component shown in Figure 3a; from top to bottom: NC_001453-NC_001627, NC_001453-NC_005929

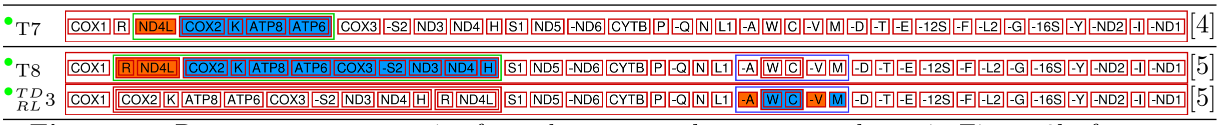

Figure 5 – Rearrangement scenarios from the connected component shown in Figure 3b; from top to bottom: NC_001878-NC_007689, NC_001878-NC_010692

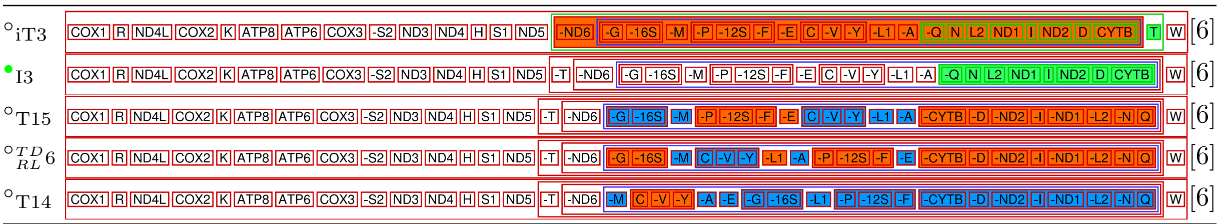

Figure 6 – Rearrangement scenario from the connected component shown in Figure 3c, i.e. NC_005930-NC_005334

1.2 Mollusca and Annelida

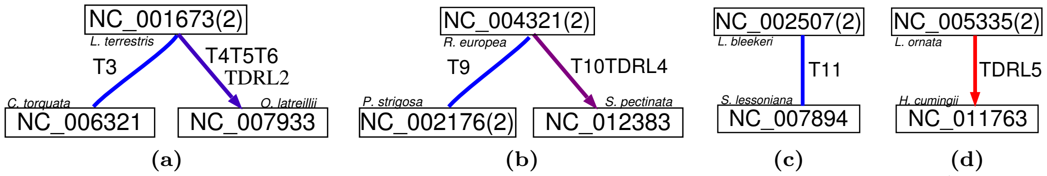

Figure 7 – Connected components of size greater than one including gene orders from a) *Annelida* and b-d) *Mollusca*; b) *Gastropoda*; c) *Cephalopoda*; d) *Bivalvia*

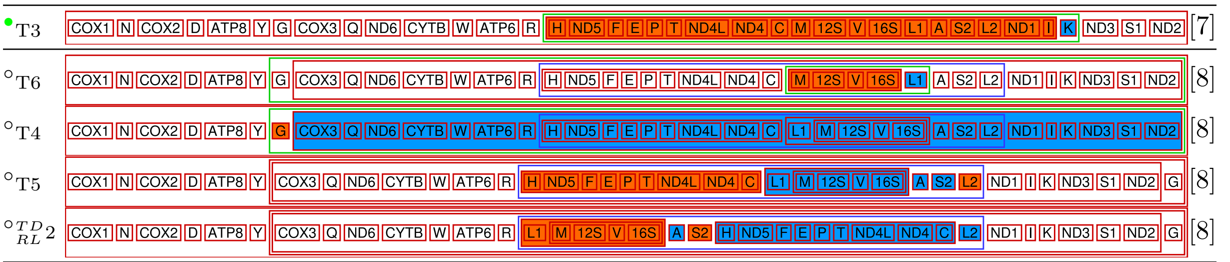

Figure 8 – Rearrangement scenarios from the connected component shown in Figure 7a; from top to bottom: NC_001673-NC_006321 and NC_001673-NC_007933

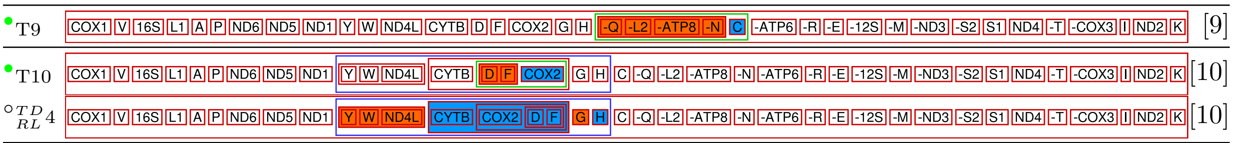

Figure 9 – Rearrangement scenarios from the connected component shown in Figure 7b; from top to bottom: NC_002176-NC_004321, NC_004321-NC_012383

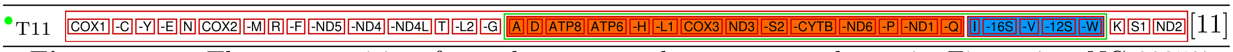

Figure 10 – The transposition from the connected component shown in Figure 7c; NC_002507-NC_007894

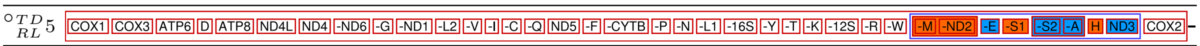

Figure 11 – TDRL from the connected component shown in Figure 7d; NC_005335-NC_011763

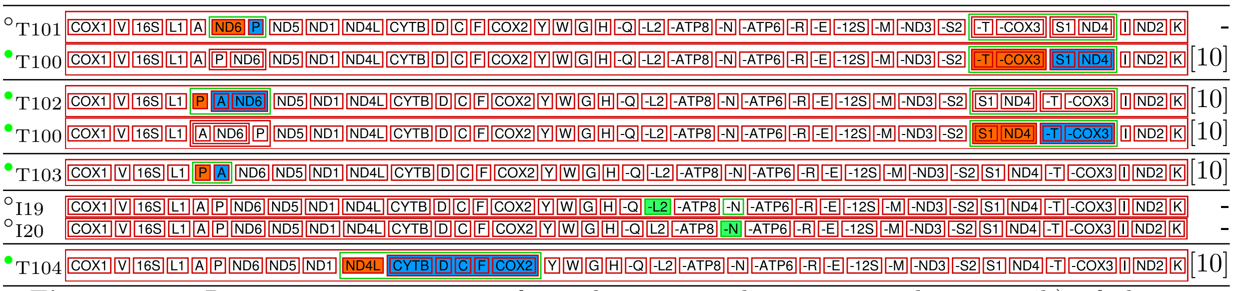

Figure 12 – Rearrangement scenarios from the connected component shown in 4b) of the main text; from top to bottom: NC_001816-NC_005439, NC_001761-NC_001816, NC_001761-NC_005439, NC_005439-NC_010220, and NC_005439-NC_012434

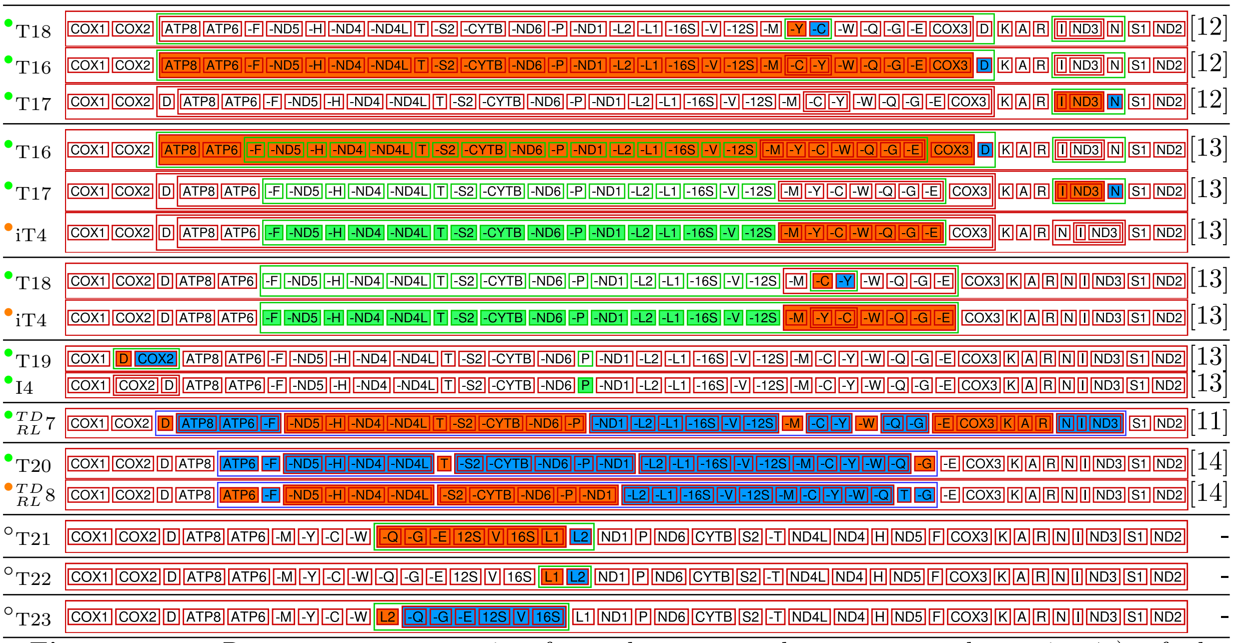

Figure 13 – Rearrangement scenarios from the connected component shown in 4a) of the main text; from top to bottom: NC_005940-NC_006353, NC_005940-NC_007781, NC_006353-NC_007781, NC_001636-NC_006353, NC_006353-NC_007895, NC_006353-NC_007980, NC_007781-NC_008797, NC_007781-NC_012899, and NC_008797-NC_012899

1.3 Arthropoda

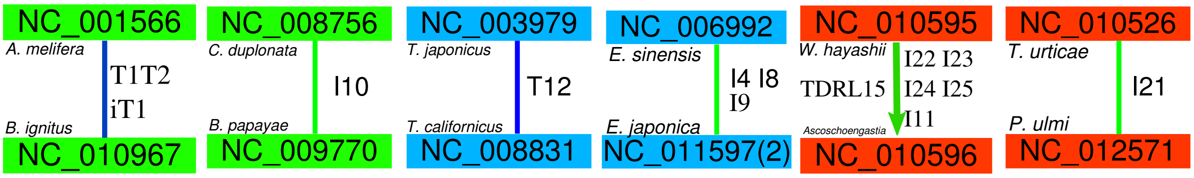

(a) (b) (c) (d) (e) (f)

Figure 14 – The connected components of size two including mitochondrial gene orders from *Arthropod* species; a,b) *Hexapoda*; c,d) *Crustacea*; e,f) *Chelicerata*

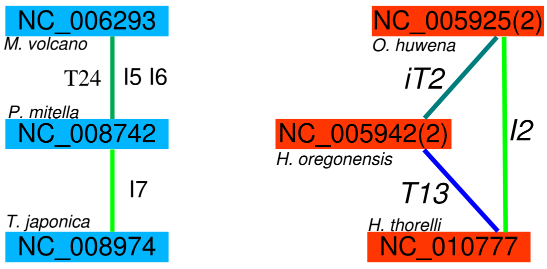

(a) (b)

Figure 15 – The connected components of size three that include mitochondrial gene orders from *Arthropod* species; a) *Crustacea*; b) *Chelicerata*

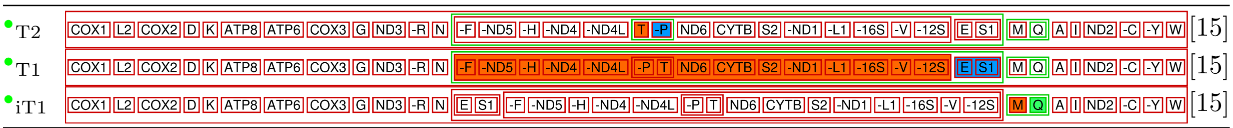

Figure 16 – Rearrangement scenario from the connected component shown in Figure 14a; NC_001566-NC_010967

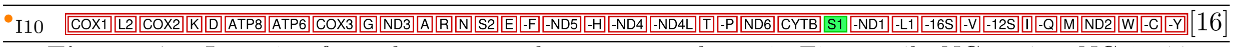

Figure 17 – Inversion from the connected component shown in Figure 14b; NC_008756-NC_009770

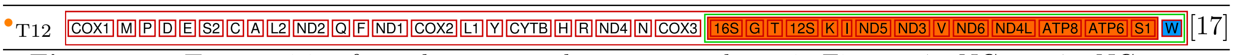

Figure 18 – Transposition from the connected component shown in Figure 14c; NC_003979-NC_008831

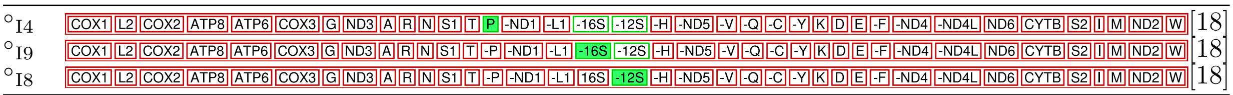

Figure 19 – Rearrangement scenario from the connected component shown in Figure 14d; NC_006992-NC_011597

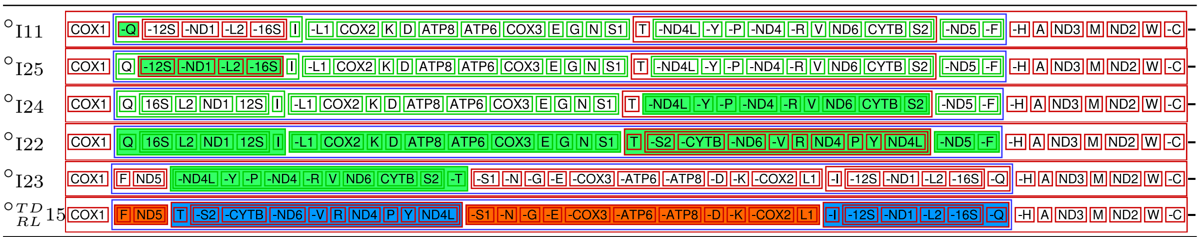

Figure 20 – Rearrangement scenario from the connected component shown in Figure 14e; NC_010595-NC_010596

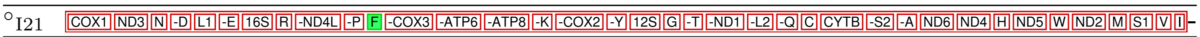

Figure 21 – Inversion from the connected component shown in Figure 14f; NC_010526-NC_012571

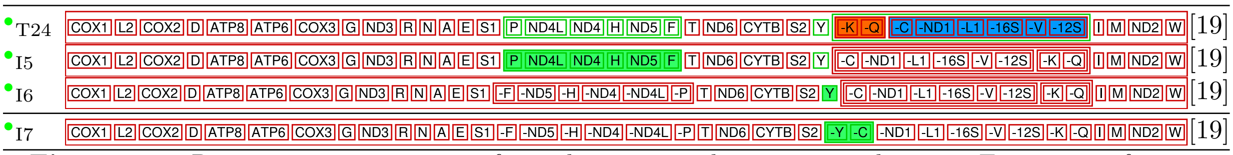

Figure 22 – Rearrangement scenarios from the connected component shown in Figure 15a; from top to bottom: NC_006293-NC_008742, NC_008742-NC_008974

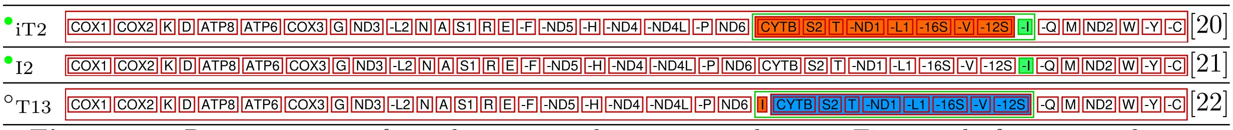

Figure 23 – Rearrangements from the connected component shown in Figure 15b; from top to bottom: NC_005925-NC_005942, NC_005925-NC_010777, and NC_005942-NC_010777

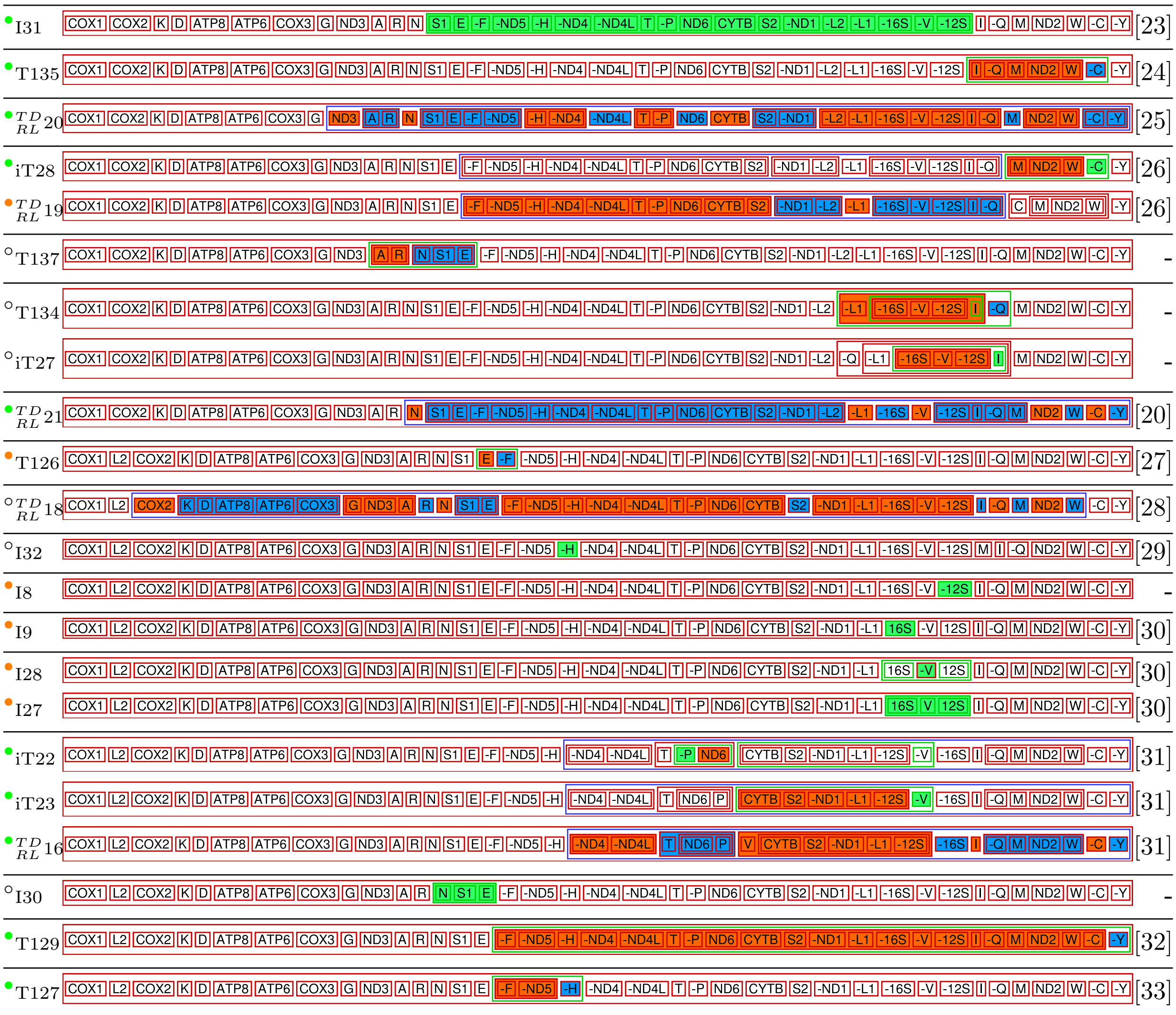

Figure 24 – Rearrangement scenarios from the connected component shown in 5 of the main text; from top to bottom: NC_002010-NC_008557, NC_002010-NC_002629, NC_002010-NC_005870, NC_002010-NC_002074, NC_002010-NC_010430, NC_002010-NC_010766, NC_002010-NC_009985, NC_000844-NC_003081, NC_000844-NC_004816, NC_002355-NC_008141, NC_000844-NC_012463, NC_006081-NC_012463, NC_006081-NC_007010, NC_007010-NC_011243, NC_000844-NC_011823, NC_000844-NC_007688, and NC_000844-NC_005037

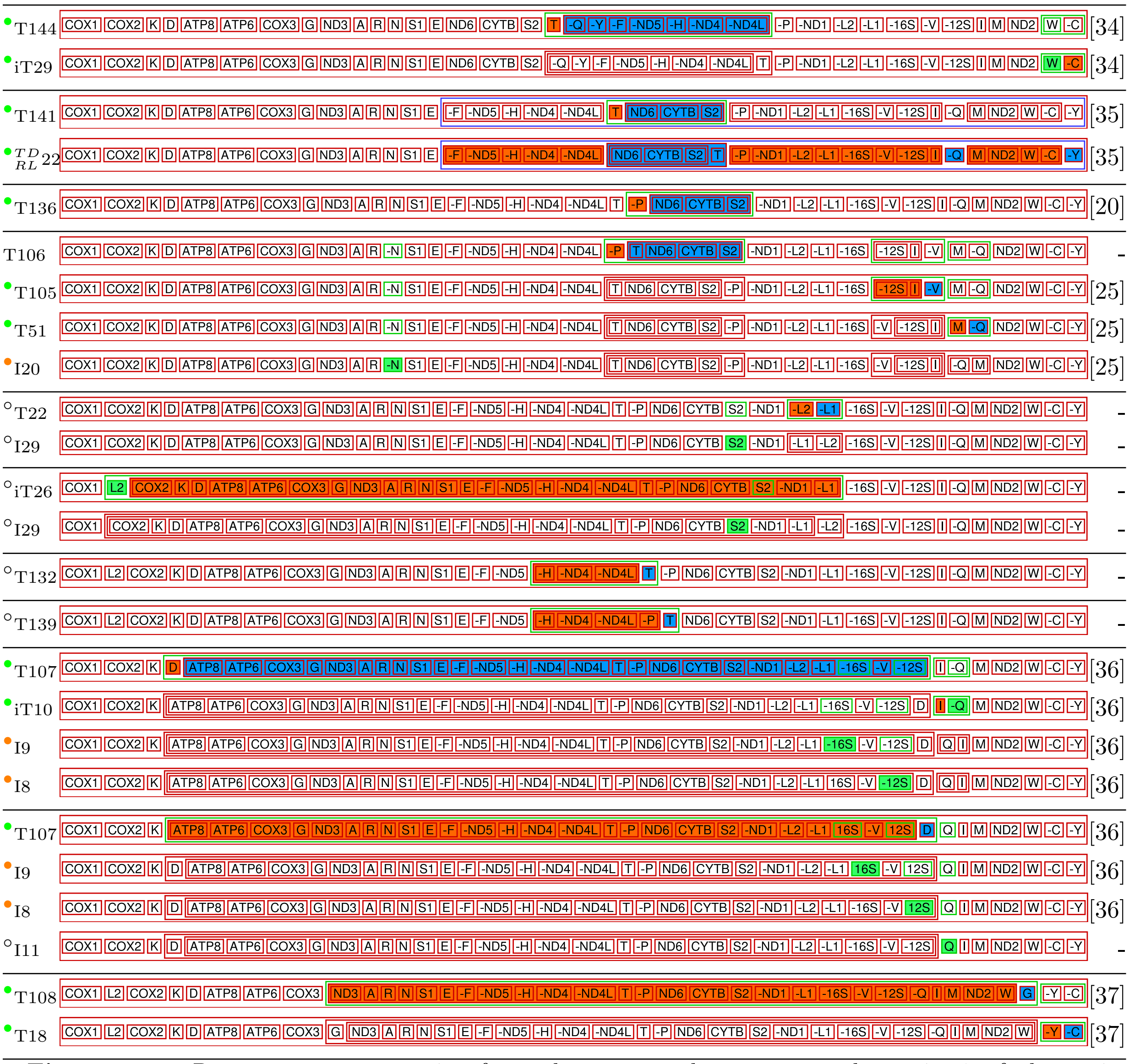

Figure 25 – Rearrangement scenarios from the connected component shown in 6 of the main text (left half); from top to bottom: NC_003343-NC_010221, NC_009984-NC_003343, NC_002010-NC_009984, NC_008453-NC_009984, NC_002010-NC_010779, NC_000844-NC_010779, NC_000844-NC_012738, NC_012421-NC_012738, NC_002010-NC_006515, NC_006515-NC_009724, and NC_006280-NC_012459

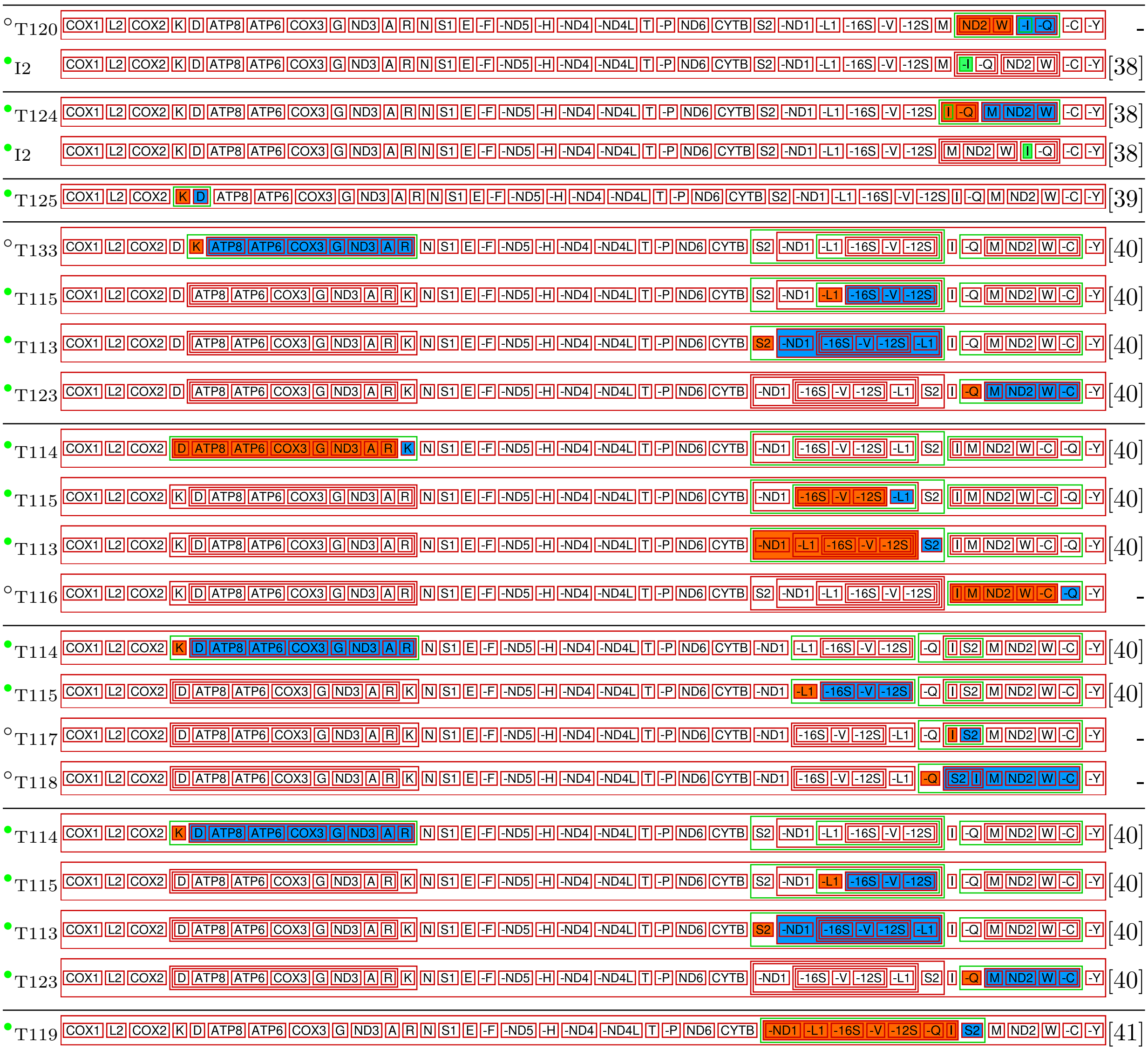

Figure 26 – Rearrangement scenarios from the connected component shown in 6 of the main text (bottom right quadrant); from top to bottom: NC_001620-NC_002355, NC_000844-NC_001620, NC_000844-NC_001712, NC_001712-NC_005934, NC_005934-NC_012459, NC_002735-NC_005934, NC_000844-NC_005934, and NC_002735-NC_012459

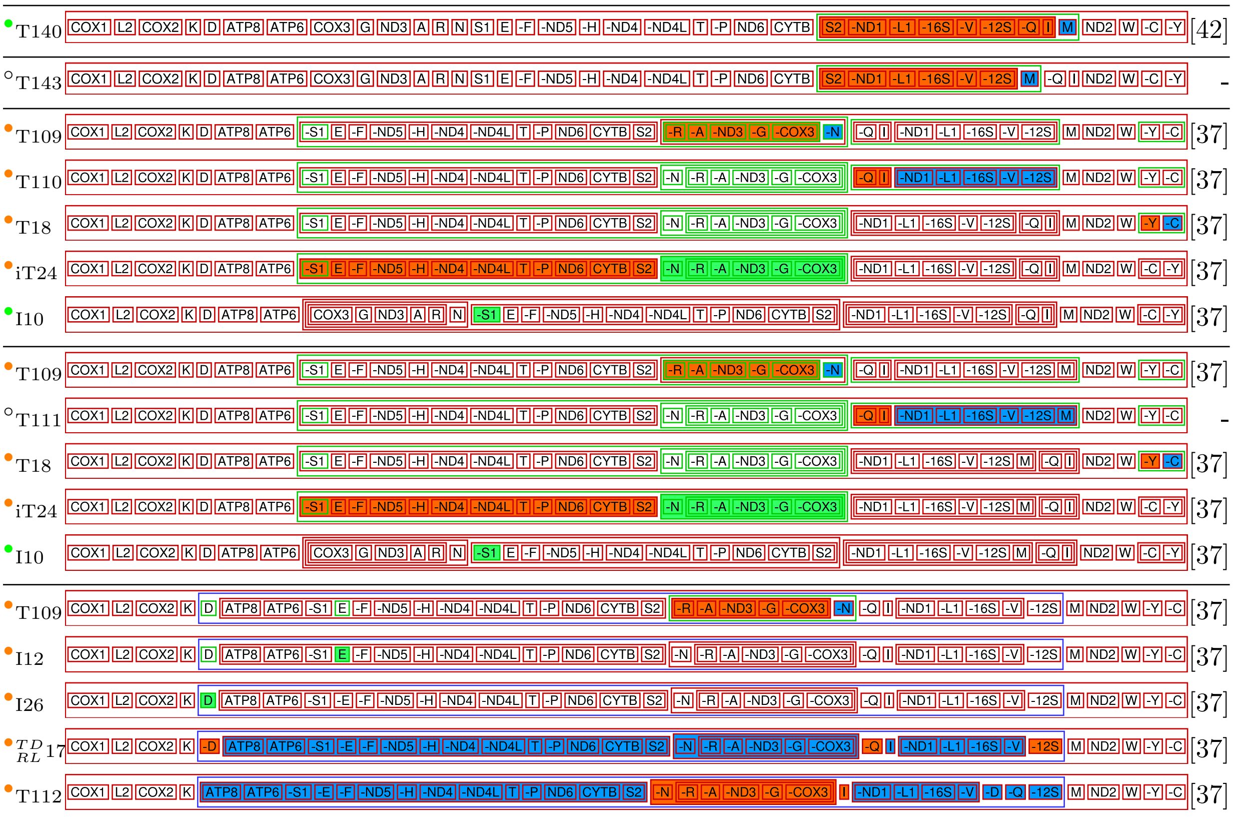

Figure 27 – Rearrangement scenarios from the connected component shown in 6 of the main text (at the bottom); from top to bottom: NC_012459-NC_012689, NC_012688-NC_012689, NC_006160-NC_012459, NC_006160-NC_012688, and NC_006160-NC_006279

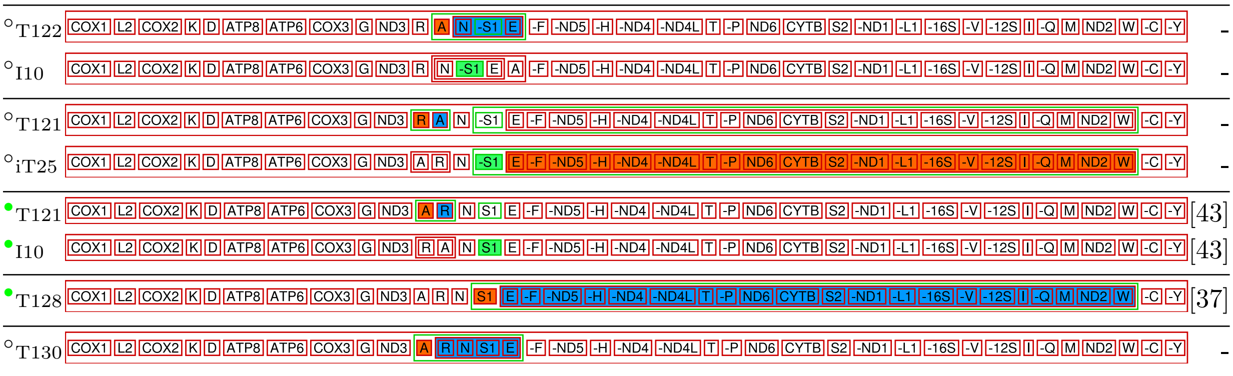

Figure 28 – Rearrangement scenarios from the connected component shown in 6 of the main text (top right quadrant); from top to bottom: NC_000875-NC_010532, NC_000875-NC_006158, NC_000844-NC_000875, NC_000844-NC_006158, and NC_000844-NC_010532

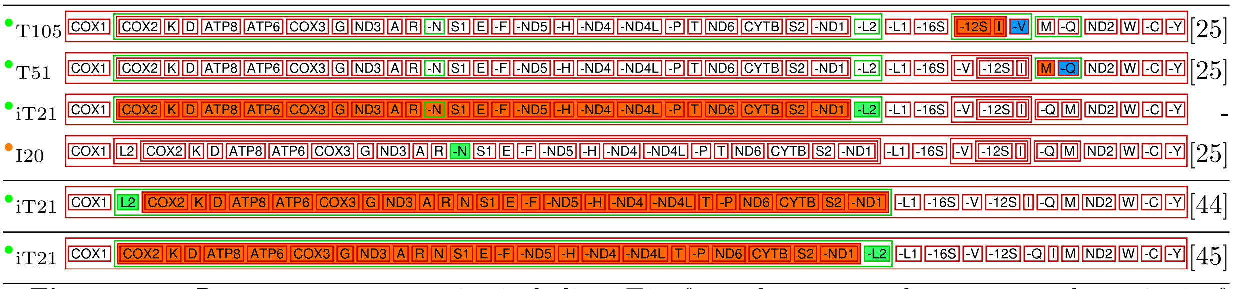

Figure 29 – Rearrangement scenarios including iT21 from the connected component shown in 6 of the main text; from top to bottom: NC_008453-NC_012421, NC_000844-NC_002010, and NC_009724-NC_012459

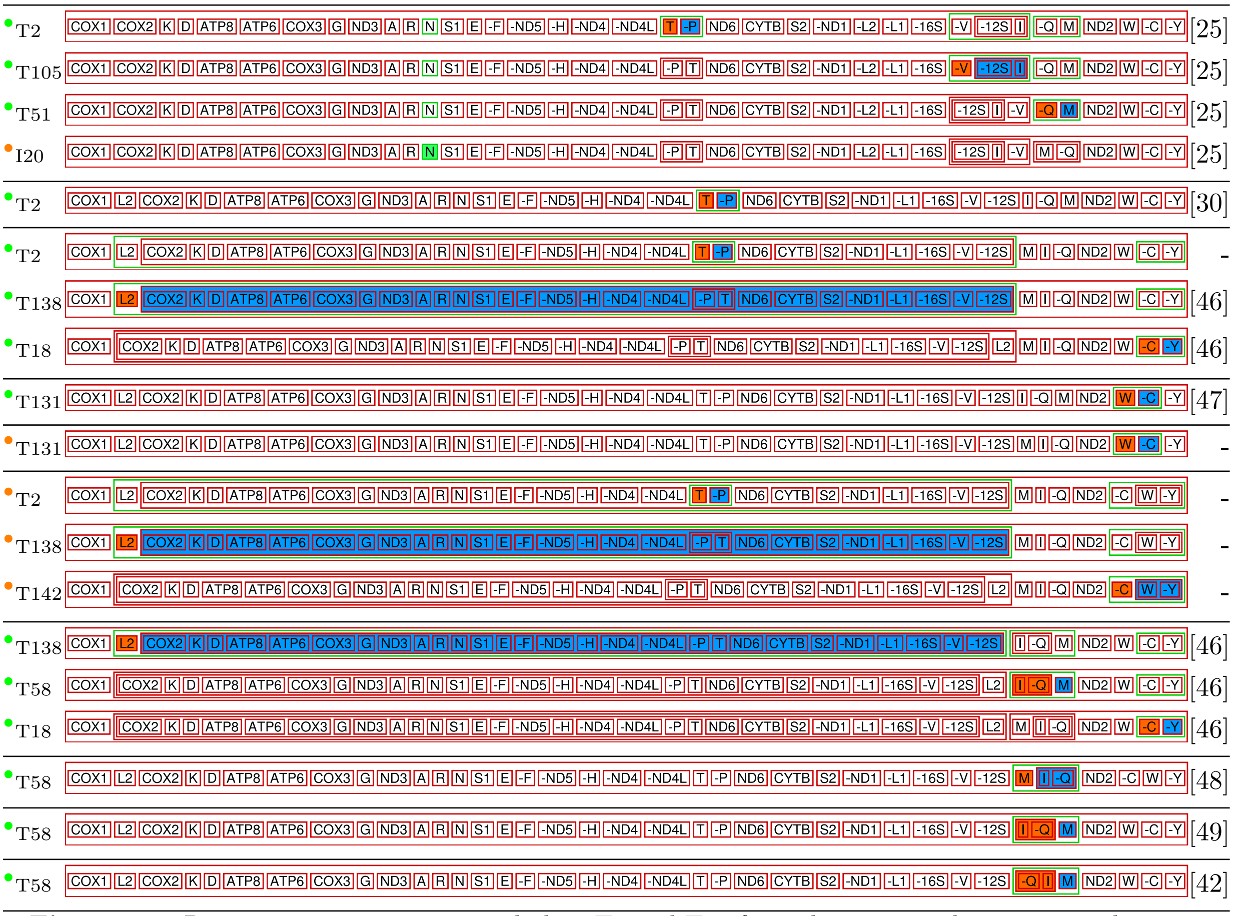

Figure 30 – Rearrangement scenarios including T2 and T58 from the connected component shown in 6 of the main text; from top to bottom: NC_002010-NC_008453, NC_000844-NC_012421, NC_002355-NC_012708, NC_000844-NC_011277, NC_002355-NC_011128, NC_011128-NC_012708, NC_012421-NC_012708, NC_011128-NC_011277, NC_000844-NC_002355, and NC_012459-NC_012688 [29]

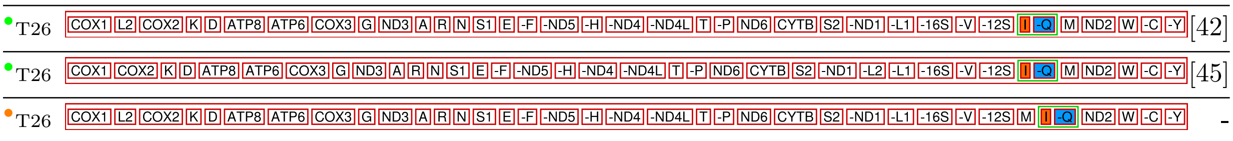

Figure 31 – Transposition 26 from the connected component shown in 6 of the main text; from top to bottom: NC_000844-NC_012459, NC_002010-NC_009724, and NC_002355-NC_012688

1.4 Chordata, Hemichordata, X. bocki

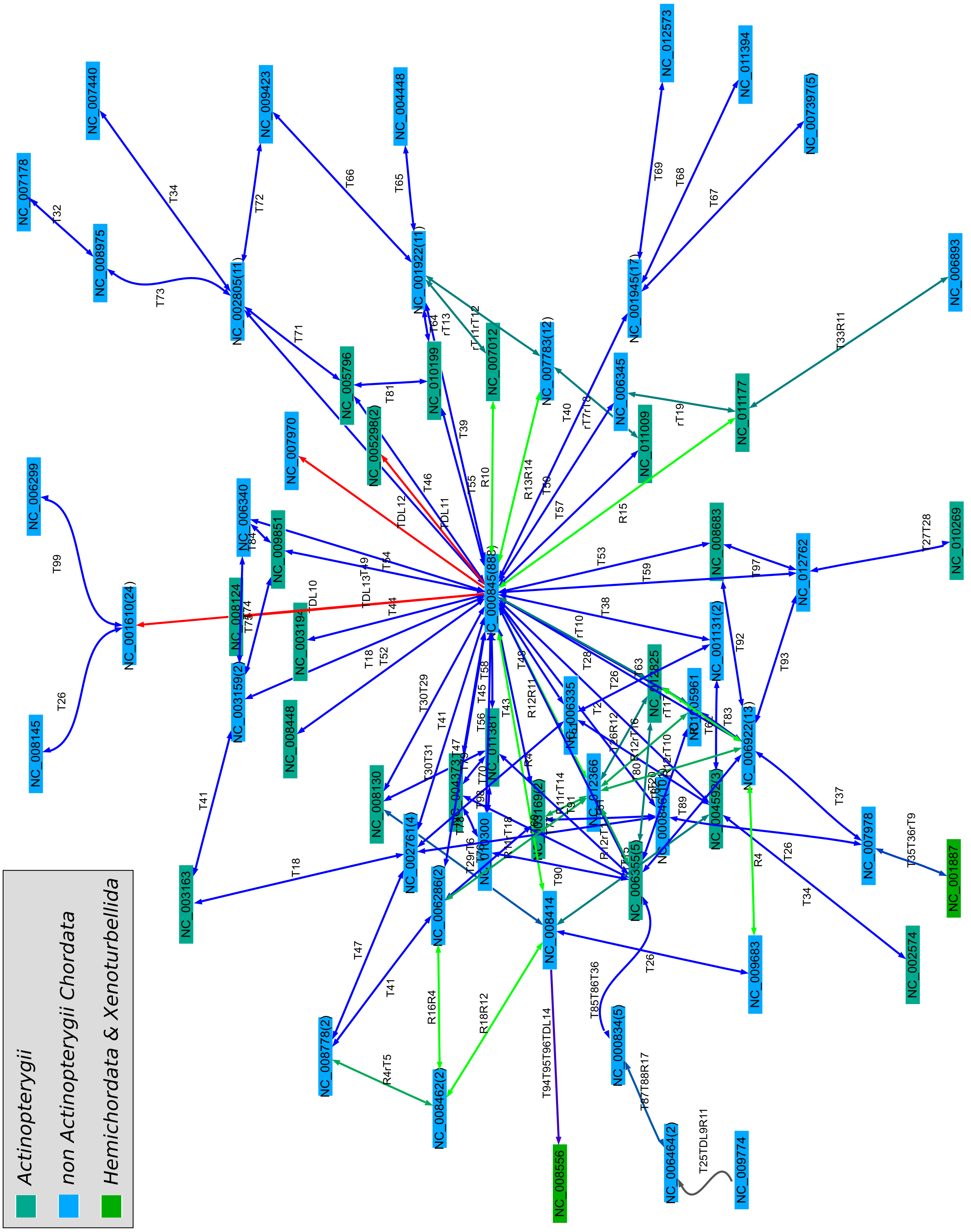

Figure 32 – The largest connected component including the mitochondrial gene orders from *Chordates* (blue), one *Hemichordata*, and *X. bocki*

1.5 CREx Operations

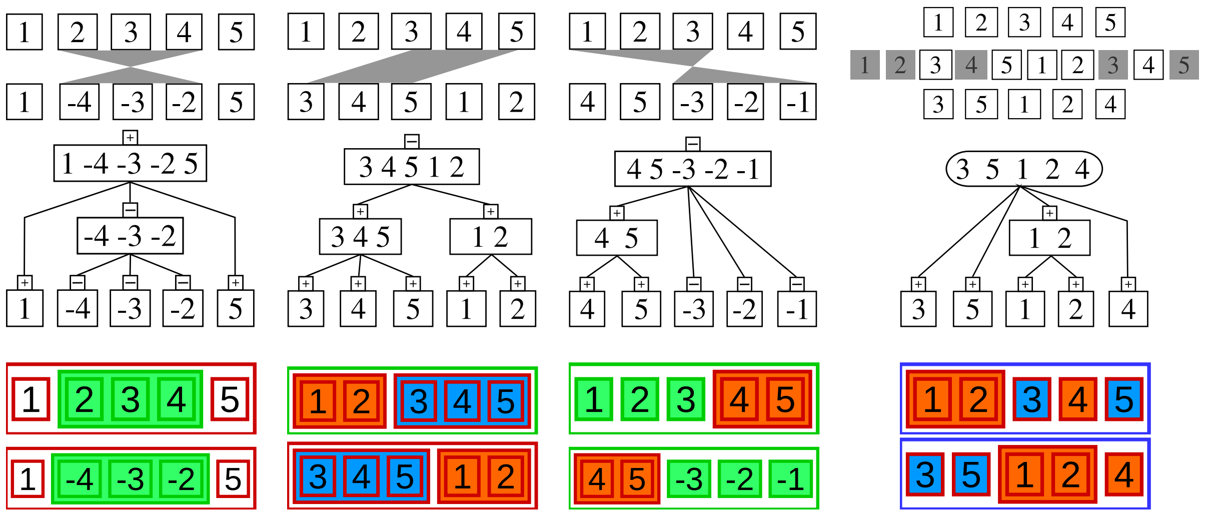

Figure 33 – Gene order rearrangement events considered in CREx; from left to right: inversion, transposition, inverse transposition, and tandem duplication random loss; top: the effects of an example rearrangement on the identity permutation of length five; middle: strong interval tree of the resulting permutation and the identity permutation; bottom: alternative representation of the strong interval tree as *family diagram* (shown with respect to identity and with respect to the resulting permutation); each box represents a strong common interval; the inclusion relation is represented by the inclusion of the boxes; the line colours of the boxes indicate node types (red: increasing, green: decreasing, and blue prime); the fill colours of the boxes indicate rearranged intervals (inversion: green, transposition: a red and a blue box mark the swapped intervals, inverse transposition: the inverse transposed interval is shown in green and the transposed part in red, TDRL: intervals kept in the first copy in blue and intervals kept in the second copy in red)

References

1. Bernt M: **Gene order rearrangement methods for the reconstruction of phylogeny**. *PhD thesis*, Universität Leipzig 2010.
2. Jacobs HT, Asakawa S, Araki T, Miura K, Smith MJ, Watanabe K: **Conserved tRNA gene cluster in starfish mitochondrial DNA.** *Curr Genet* 1989, **15**(3):193–206.
3. Arndt A, Smith MJ: **Mitochondrial gene rearrangement in the sea cucumber genus Cucumaria.** *Mol Biol Evol* 1998, **15**(8):9–16.
4. Scouras A, Smith MJ: **The complete mitochondrial genomes of the sea lily Gymnocrinus richeri and the feather star Phanogenia gracilis: signature nucleotide bias and unique nad4L gene rearrangement within crinoids.** *Mol Phylogenet Evol* 2006, **39**(2):323–334.
5. Perseke M, Fritzsch G, Ramsch K, Bernt M, Merkle D, Middendorf M, Bernhard D, Stadler PF, Schlegel M: **Evolution of Mitochondrial Gene Orders in Echinoderms**. *Mol Phylogenet Evol* 2008, **47**(2):855–864.
6. Scouras A, Beckenbach K, Arndt A, Smith MJ: **Complete mitochondrial genome DNA sequence for two ophiuroids and a holothuroid: the utility of protein gene sequence and gene maps in the analyses of deep deuterostome phylogeny.** *Mol Phylogenet Evol* 2004, **31**:50–65.
7. Jennings RM, Halanych KM: **Mitochondrial Genomes of Clymenella torquata (Maldanidae) and Riftia pachyptila (Siboglinidae): Evidence for Conserved Gene Order in Annelida**. *Mol Biol Evol* 2005, **22**(2):210–222.
8. Bleidorn C, Podsiadlowski L, Bartolomaeus T: **The complete mitochondrial genome of the orbiniid polychaete Orbinia latreillii (Annelida, Orbiniidae)– A novel gene order for Annelida and implications for annelid phylogeny**. *Gene* 2006, **370**:96–103.
9. Kurabayashi A, Ueshima R: **Complete Sequence of the Insect Mol Biol of the Primitive Opisthobranch Gastropod Pupa strigosa: Systematic Implication of the Genome Organization**. *Mol Biol Evol* 2000, **17**(2):266–277.

10. Grande C, Templado J, Zardoya R: **Evolution of gastropod mitochondrial genome arrangements**. *BMC Evol Biol* 2008, **8**:61.

11. Akasaki T, Nikaido M, Tsuchiya K, Segawa S, Hasegawa M, Okada N: **Extensive mitochondrial gene arrangements in coleoid Cephalopoda and their phylogenetic implications**. *Mol Phylogenet Evol* 2006, **38**(3):648–658.

12. Maynard BT, Kerr LJ, McKiernan JM, Jansen ES, Hanna PJ: **Mitochondrial DNA Sequence and Gene Organization in Australian Backup Abalone Haliotis Rubra (Leach)**. *Marine Biotechnology* 2005, **7**(6):645–658.

13. Bandyopadhyay PK, Stevenson BJ, Cady MT, Olivera BM, Wolstenholme DR: **Complete mitochondrial DNA sequence of a Conoidean gastropod, Lophiotoma (Xenuroturris) cerithiformis: Gene order and gastropod phylogeny**. *Toxicon* 2006, **48**:29–43.

14. Boore JL: **The complete sequence of the mitochondrial genome of Nautilus macromphalus (Mollusca: Cephalopoda)**. *BMC Genomics* 2006, **7**:182.

15. Cha SY, Yoon HJ, Lee EM, Yoon MH, Hwang JS, Jin BR, Han YS, Kim I: **The complete nucleotide sequence and gene organization of the mitochondrial genome of the bumblebee, Bombus ignitus (Hymenoptera: Apidae)**. *Gene* 2007, **392**(1-2):206–220.

16. Cameron SL, Lambkin CL, Barker SC, Whiting MF: **A mitochondrial genome phylogeny of Diptera: whole genome sequence data accurately resolve relationships over broad timescales with high precision**. *Syst Entomol* 2006, **32**:40–59.

17. Burton RS, Byrne RJ, Rawson PD: **Three divergent mitochondrial genomes from California populations of the copepod Tigriopus californicus**. *Gene* 2007, **403**(1-2):53–59.

18. Sun H, Zhou K, Song D: **Mitochondrial genome of the Chinese mitten crab Eriocheir japonica sinenesis (Brachyura: Thoracotremata: Grapsoidea) reveals a novel gene order and two target regions of gene rearrangements**. *Gene* 2005, **349**:207–217.

19. Lim JT, Hwang UW: **The Complete Mitochondrial Genome of Pollicipes mitella (Crustacea, Maxillopoda, Cirripedia): Non-Monophylies of Maxillopoda and Crustacea**. *Mol Cells* 2006, **22**(3):314–322.

20. Fahrein K, Talarico G, Braband A, Podsiadlowski L: **The complete mitochondrial genome of Pseudocellus pearsei (Chelicerata: Ricinulei) and a comparison of mitochondrial gene rearrangements in Arachnida**. *BMC Genomics* 2007, **8**:386.

21. Qiu Y, Song D, Zhou K, Sun H: **The Mitochondrial Sequences of Heptathela hangzhouensis and Ornithoctonus huwena Reveal Unique Gene Arrangements and Atypical tRNAs**. *J Mol Evol* 2005, **60**:57–71.

22. Masta SE, Boore JL: **The Complete Mitochondrial Genome Sequence of the Spider Habronattus oregonensis Reveals Rearranged and Extremely Truncated tRNAs**. *Mol Biol Evol* 2004, **21**(5):893–902.

23. Webster BL, Copley RR, Jenner RA, Mackenzie-Dodds JA, Bourlat SJ, Rota-Stabelli O, Littlewood DTJ, Telford MJ: **Mitogenomics and phylogenomics reveal priapulid worms as extant models of the ancestral Ecdysozoan**. *Evol Dev* 2006, **8**(6):502–510.

24. Hwang UW, Friedrich M, Tautz D, Park CJ, Kim W: **Mitochondrial protein phylogeny joins myriapods with chelicerates**. *Nature* 2001, **413**(6852):154–157.

25. Podsiadlowski L, Kohlhagen H, Koch M: **The complete mitochondrial genome of Scutigerella causeyae (Myriapoda: Symphyla) and the phylogenetic position of Symphyla**. *Mol Phylogenet Evol* 2007, **45**:251–260.

26. Black IV WC, Roehrdanz RL: **Mitochondrial gene order is not conserved in arthropods: prostriate and metastriate tick mitochondrial genomes**. *Mol Biol Evol* 1998, **15**(12):1772–1785.

27. Friedrich M, Muqim N: **Sequence and phylogenetic analysis of the complete mitochondrial genome of the flour beetle Tribolium castanaeum**. *Mol Phylogenet Evol* 2003, **26**(3):502–512.

28. Shao R, Campbell NJH, Schmidt ER, Barker SC: **Increased Rate of Gene Rearrangement in the Mitochondrial Genomes of Three Orders of Hemipteroid Insects**. *Mol Biol Evol* 2001, **18**(9):1828–1832.

29. Lee ES, Shin KS, Kim MS, Park H, Cho S, Kim CB: **The mitochondrial genome of the smaller tea tortrix Adoxophyes honmai (Lepidoptera: Tortricidae)**. *Gene* 2006, **373**:52–57.

30. Hua J, Li M, Dong P, Cui Y, Xie Q, Bu W: **Comparative and phylogenomic studies on the mitochondrial genomes of Pentatomomorpha (Insecta: Hemiptera: Heteroptera)**. *BMC Genomics* 2008, **9**:610.

31. Miller AD, Nguyen TTT, Burridge CP, Austin CM: **Complete mitochondrial DNA sequence of the Australian freshwater crayfish, Cherax destructor (Crustacea: Decapoda: Parastacidae): a novel gene order revealed**. *Gene* 2004, **331**:65–72.

32. Podsiadlowski L: **The mitochondrial genome of the bristletail Petrobius brevistylis (Archaeognatha: Machilidae)**. *Insect Mol Biol* 2006, **15**(3):253–258.

33. Yamauchi MM, Miya MU, Nishida M: **Complete mitochondrial DNA sequence of the swimming crab, Portunus trituberculatus (Crustacea: Decapoda: Brachyura)**. *Gene* 2003, **311**:129–135.

34. Woo HJ, Lee YS, Park SJ, Lim JT, Jang KH, Choi EH, Choi YG, Hwang UW: **Complete Mitochondrial Genome of a Troglobite Millipede Antrokoreana gracilipes (Diplopoda, Juliformia, Julida), and Juliformian Phylogeny**. *Mol Cells* 2007, **23**(2):182–191.

35. Lavrov DV, Boore JL, Brown WM: **Complete mtDNA Sequences of Two Millipedes Suggest a New Model for Mitochondrial Gene Rearrangements: Duplication and Nonrandom Loss**. *Mol Biol Evol* 2002, **19**(2):163–169.

36. Dávila S, Piñero D, Bustos P, Cevallos MA, Dávila G: **The mitochondrial genome sequence of the scorpion Centruroides limpidus (Karsch 1879) (Chelicerata; Arachnida)**. *Gene* 2005, **360**(2):92–102.

37. Thao ML, Baumann L, Baumann P: **Organization of the mitochondrial genomes of whiteflies, aphids, and psyllids (Hemiptera, Sternorrhyncha)**. *BMC Evol Biol* 2004, **4**:25.

38. Valverde JR, Batuecas B, Moratilla C, Marco R, Garesse R: **The complete mitochondrial DNA sequence of the crustacean Artemia franciscana**. *J Mol Evol* 1994, **39**(4):400–408.

39. Flook PK, Rowell CHF, Gellissen G: **The sequence, organization, and evolution of the Locusta migratoria mitochondrial genome**. *J Mol Evol* 1995, **41**(6):928–941.

40. Lavrov DV, Brown WM, Boore JL: **Phylogenetic position of the Pentastomida and (pan)crustacean relationships**. *Proc Roy Soc B* 2004, **271**(1538):537–544.

41. Nardi F, Carapelli A, Fanciulli PP, Dallai R, Frati F: **The Complete Mitochondrial DNA Sequence of the Basal Hexapod Tetrodontophora bielanensis: Evidence for Heteroplasmy and tRNA Translocations**. *Mol Biol Evol* 2001, **18**(7):1293–1304.

42. Dowton M, Cameron SL, Dowavic JI, Austin AD, Whiting M: **Characterization of 67 Mitochondrial tRNA Gene Rearrangements in the Hymenoptera Suggests That Mitochondrial tRNA Gene Position Is Selectively Neutral**. *Mol Biol Evol* 2009, **26**(7):1607–1617.

43. Beard CB, Hamm DM, Collins FH: **The mitochondrial genome of the mosquito Anopheles gambiae: DNA sequence, genome organization, and comparisons with mitochondrial sequences of other insects**. *Insect Mol Biol* 1993, **2**(2):103–124.

44. Boore JL, Lavrov DV, Brown WM: **Gene translocation links insects and crustaceans**. *Nature* 1998, **392**(6677):667–668.

45. Park SJ, Lee YS, Hwang UW: **The complete mitochondrial genome of the sea spider Achelia bituberculata (Pycnogonida, Ammotheidae): arthropod ground pattern of gene arrangement**. *BMC Genomics* 2007, **8**:343.

46. Wei SJ, M S, H HJ, Sharkey M, Chen XX: **The complete mitochondrial genome of Diadegma semiclausum (Hymenoptera: Ichneumonidae) indicates extensive independent evolutionary events**. *Genome* 2009, **52**(4):308–319.

47. Beckenbach AT, Stewart JB: **Insect mitochondrial genomics 3: the complete mitochondrial genome sequences of representatives from two neuropteroid orders: a dobsonfly (order Megaloptera) and a giant lacewing and an owlfly (order Neuroptera)**. *Genome* 2009, **52**:31–38.

48. Salvato P, Simonato M, Battisti A, Negrisolo E: **The complete mitochondrial genome of the bag-shelter moth Ochrogaster lunifer (Lepidoptera, Notodontidae)**. *BMC Genomics* 2008, **9**:331.

49. Taylor MF, McKechnie SW, Pierce N, Kreitman M: **The lepidopteran mitochondrial control region: structure and evolution**. *Mol Biol Evol* 1993, **10**(6):1259–1272.
